# Radon-Induced Radiation Biomarkers: A Scoping Review from Exposure Dosimetry to Early Biological Effects on the Lung

**DOI:** 10.3390/ijms27104391

**Published:** 2026-05-14

**Authors:** Phoka C. Rathebe, Mota Kholopo

**Affiliations:** Department of Environmental Health, Faculty of Health Sciences, Doornfontein Campus, University of Johannesburg, P.O. Box 524, Johannesburg 2006, South Africa; motaliok@gmail.com

**Keywords:** radon-222, alpha particles, biomarkers, miRNA, lung cancer

## Abstract

Radon-222, a naturally occurring radioactive gas, is the second leading cause of lung cancer globally, after tobacco use. When inhaled, its decay products, especially polonium-218 and polonium-214, emit high-energy alpha particles that induce dense DNA damage in the bronchial epithelium. Because ambient radon measurements often vary significantly over time and across locations, they provide limited insight into individual exposure levels. This suggests the urgent need for biological markers that can accurately indicate internal dose and early signs of lung cancer development. This review offers an extensive overview of biomarkers associated with radon exposure, from internal dosimetry to early biological responses. It covers internal dose markers (e.g., radon progeny in air and ^210^Po/^210^Pb in bones and teeth), molecular and cytogenetic indicators of effective dose (such as chromosomal aberrations, γ-H2AX foci, and DNA adducts), and early effect markers (including somatic mutations, epigenetic changes, miRNA profiles, and autoantibody signatures). The review highlights translocations detected via FISH, discussing those that are stable over time versus those that are transient. It also evaluates the reliability and practicality of these biomarkers in occupational and residential settings, noting how smoking complicates causal inference due to overlapping mutation pathways. Finally, it suggests that integrating multi-omics technologies could improve the precision of biomarker panels.

## 1. Introduction

Radon-222 is a colorless, odorless radioactive gas produced by the natural decay of uranium in soil and rock. It has become a significant environmental carcinogen with widespread effects on global public health. Humans mainly encounter radon through inhalation, especially indoors, where it can build up to dangerous levels. Although it occurs naturally, radon is a subtle and often overlooked hazard, now acknowledged worldwide as the second leading cause of lung cancer after tobacco use and the top cause among never-smokers. Recent epidemiological data highlights the significant role of radon in the global lung cancer burden. A 2022 global assessment by Shan et al. [1] estimated that lung cancer deaths due to residential radon exposure amounted to around 1.03 per 100,000 people, accounting for approximately 84,000 deaths annually worldwide. These numbers reveal a troubling ongoing impact of radon-related health issues, especially in areas with high radon levels and limited mitigation infrastructure. In the United States, the Environmental Protection Agency (EPA) estimates that about 21,000 lung cancer deaths each year are caused by radon exposure [2]. This figure surpasses other commonly discussed hazards like secondhand smoke, drunk driving, or fire-related fatalities. Notably, many of these deaths happen among people who have never smoked, highlighting radon’s ability to act as a powerful and independent carcinogen. The World Health Organization (WHO) has repeatedly highlighted these issues, stressing that radon-related lung cancers are preventable. Differences in exposure across regions are mainly caused by geological factors like granitic bedrock, as well as building materials and ventilation norms.

The population attributable fraction (PAF) offers an essential epidemiological perspective on how radon impacts public health. PAF estimates the percentage of disease cases that could be prevented if the risk factor, such as radon in this context, were eliminated. Worldwide, radon is believed to contribute to 3% to 14% of all lung cancer deaths, with the highest estimates observed in regions with high radon levels, including parts of Central Europe, North America, and Scandinavia [3]. The synergistic (more precisely, multiplicative) relationship between radon exposure and tobacco smoking increases risk. Smokers exposed to radon have a 10 to 20 times higher chance of developing lung cancer than never-smokers exposed to the same radon levels [4]. Although the absolute risk for never-smokers is lower, the public health impact on this group is still significant because they face fewer other carcinogenic exposures. In Canada, it was estimated that 6.9% of lung cancers in 2015, amounting to 1741 cases, were caused by radon, with numbers expected to rise if current exposure levels continue [5]. Notably, many of these cases would happen in homes where radon levels are below national regulatory thresholds, indicating that even lower levels might still pose significant risks.

Radon risk distribution is not uniform; it is graded across residential and occupational areas, each with its own vulnerabilities. Regarding occupational settings, some of the strongest epidemiological evidence linking radon to lung cancer comes from cohort studies of miners exposed to high levels of radon decay products in underground uranium and tin mines. Seminal findings from the Colorado Plateau uranium miners and Chinese tin miners established radon as a definitive human carcinogen. More recent research continues to highlight the importance of occupational exposures. A 2020 Canadian study estimated that 0.8% of lung cancer cases, about 188 annually, can be attributed to radon exposure at work, especially among indoor workers and those in underground jobs. Outside of occupational settings, radon levels in residential settings are greatly influenced by local geology and building characteristics. Homes built on uranium-rich soils, granite bedrock, or glacial deposits are particularly susceptible to radon entry. Factors such as inadequate sub-slab ventilation, cracks in foundations and basements, and energy-efficient insulation can worsen radon accumulation. In Korea, where the average radon level in detached houses was 116.4 Bq/m^3^, the estimated population attributable fraction (PAF) for lung cancer caused by residential radon was 6.6% for men and 4.7% for women [6]. To address this widespread threat, global health authorities have set reference levels, which are thresholds where mitigation is strongly advised. These are not necessarily safety limits but practical benchmarks. The World Health Organization (WHO) recommends a reference level of 100 Bq/m^3^, with an upper limit of 300 Bq/m^3^ when achieving the lower level is difficult [7]. The U.S. Environmental Protection Agency (US EPA) has an action level of 148 Bq/m^3^ (4 pCi/L), recommending remedial measures such as sub-slab depressurization and improved ventilation for homes above this level [2]. Under the 2013 Euratom Directive, the European Union requires member states to establish national reference levels not exceeding 300 Bq/m^3^ [8,9]. These guidelines differ due to practical constraints like technological capacity and economic factors, but all highlight one key point: radon is a preventable and manageable carcinogen.

The radiobiological hazard of radon-222 (^222^Rn) stems not from the gas itself but from its short-lived solid progeny, especially polonium-218 (^218^Po) and polonium-214 (^214^Po). These emit high-LET alpha particles that can cause significant DNA damage in lung tissue. Although ^222^Rn is a chemically inert noble gas, its decay triggers a cascade within the uranium-238 (^238^U) series, rapidly transitioning through alpha and beta-emitting isotopes, including ^218^Po, ^214^Pb, ^214^Bi, and ^214^Po, before reaching the long-lived isotope ^210^Pb. These alpha-emitting progeny emit densely ionizing radiation over a short range (40–70 µm in tissue), making them biologically potent. Their half-lives (3.05 min for ^218^Po and only 164 microseconds for ^214^Po) mean most decay occurs during inhalation or shortly after deposition in the respiratory tract. When formed, these progenies are electrically charged and rapidly attach to airborne particles (aerosols), although some remain unattached. The unattached fraction is especially hazardous; its ultrafine size and high diffusivity enable deep penetration into the bronchial epithelium, where alpha decay deposits highly localized radiation doses in basal and secretory cells, which are critical targets for carcinogenesis [10]. To quantify exposure risk, especially in occupational settings, historical units such as working levels (WLs) and working level months (WLMs) have been established [11]. One WL indicates the alpha energy emitted by short-lived radon progeny in equilibrium with 100 pCi/L (~3700 Bq/m^3^) of ^222^Rn [12,13]. One WLM equals exposure to 1 WL for 170 h (the number of working hours in a month), serving as a crucial metric in miner epidemiology studies that form the basis of current radon risk models [12,14]. Although modern studies measure radon in Bq/m^3^, its biological relevance still depends on the behavior and decay of its progeny. The physicochemical properties, deposition efficiency, and dose-delivery profiles of these progenies are the main factors in radon-induced lung cancer, emphasizing the need for biomarker research that can identify early effects from this localized and potent radiation source [15,16,17].

### Radon Dosimetry and Biomarkers: From Exposure to Impact

The pathway from environmental radiation to lung cancer starts with inhaling radon-222, a chemically inert gas that is mostly exhaled unchanged. The main radiological risk comes from its short-lived alpha-emitting decay products, particularly polonium-218 and polonium-214, which decay in the respiratory tract and deliver highly localized, high-linear energy transfer (LET) radiation [18,19]. These solid decay products exist in two physical states: an unattached fraction, made of ultrafine particles (<5 nm) that deposit efficiently in the tracheobronchial region via Brownian diffusion, and an attached fraction, bound to larger ambient aerosols that can penetrate deeper into the alveoli or be exhaled [10]. Notably, the unattached fraction is more significant for dosimetry because its deposition overlaps with the basal and secretory cells of the bronchial epithelium, which are involved in tissue regeneration and are key targets for malignant transformation [10,19]. When alpha particles decay near or inside the epithelial lining, their brief path (40–70 µm) causes energy to directly target the cell nuclei. Unlike low-LET radiation, alpha particles create complex, clustered DNA damage such as double-strand breaks, base lesions, and DNA-protein crosslinks, often within just a few turns of the DNA helix [20]. These types of damage are not only hard to repair but also likely to be misrepaired, increasing the risk of chromosomal abnormalities and mutations, which are key indicators of cancer development [21,22]. Despite this well-characterized dosimetric pathway, current radon risk assessments predominantly depend on environmental measurements, especially air concentration in B/m^3^, as the main exposure metric. However, these measurements are indirect and constrained by spatial variability, such as differences between rooms or floors; temporal fluctuations, including diurnal and seasonal changes; and a lack of behavioral data, like time spent indoors or breathing patterns. These limitations hinder accurate exposure estimation and lead to an underestimation of the true biological dose to the lungs [23,24]. Biomarkers provide a compelling solution to these challenges [25]. Internal dose indicators like lead-210 (^210^Pb) in bones or teeth accumulate over time, reflecting lifetime radiation exposure regardless of location or behavior. More crucially, biomarkers of biologically effective dose, such as chromosomal aberrations, γ-H2AX foci, or oxidative DNA damage, reveal the actual interaction between radiation and cellular targets [22]. Additionally, early effect indicators (including gene mutations, microRNA profile changes, and epigenetic modifications) offer insights into the initial biological alterations that occur before the development of cancer [19,26].

The review expands on the initial rationale, providing a detailed and critical synthesis of radon-induced biomarkers. It is organized along a biological continuum, from internal dose and molecular damage to functional cellular responses. The review aims to (1) catalogue and assess both well-known and emerging biomarkers; (2) investigate mechanistic links between alpha-particle exposure and distinct molecular signatures; (3) evaluate the role of multi-omics and high-throughput techniques in biomarker discovery; and (4) consider methodological factors such as smoking as a confounder, tissue-specific differences, and individual variability. By bridging radiation physics with molecular epidemiology, this review aims to highlight a translational pathway from environmental exposure to practical insights, ultimately promoting precision prevention and early detection of radon-induced lung cancer. To ensure the review offers a comprehensive and evidence-based overview of current knowledge, this section outlines the structured approach used to identify, select, and analyze relevant literature on radon-related biomarkers across molecular mechanistic domains.

## 2. Methodology

The protocol for this scoping review has not been registered on any registration platforms. To ensure comprehensive and transparent coverage of the scientific evidence, a structured literature search was carried out across three major databases: PubMed, Scopus, and Web of Science. The search strategy used key terms combined with Boolean operators relating to radon exposure and biological responses, including “Radon,” “Alpha radiation,” “biomarker,” “DNA damage,” “oxidative stress,” “epigenetic,” “MicroRNA,” and “lung cancer”. The search was limited to peer-reviewed articles published in English from 1990 to 2025, covering both experimental and epidemiological studies that provided molecular, cytogenetic, or omics-based evidence of biological effects linked to radon or its progeny. Additional relevant studies were identified by manually reviewing the reference lists of key papers and authoritative reports from international organizations such as the World Health Organization (WHO), the International Commission on Radiological Protection (ICRP), and the United States Environmental Protection Agency (EPA). This approach ensured the inclusion of essential foundational literature and policy-relevant evidence frequently cited in radiation health sciences. Studies were included if they presented measurable biomarkers related to internal dose, biologically effective dose, or early biological effects of radiation exposure, or if they contributed mechanistic or methodological insights relevant to biomarker discovery and validation. Both human and experimental studies were considered, provided that the exposure conditions were clearly characterized. Studies that focus exclusively on environmental or geological measurements without biological endpoints were excluded. All retrieved records were screened by title and abstract to assess relevance, followed by a detailed review of full-text publications that met eligibility criteria. The evidence was synthesized narratively rather than through quantitative pooling, due to the heterogeneity in study designs, exposure metrics, and biomarker endpoints. This structured narrative method enhances transparency and reproducibility in the review, offering a clear, mechanistically based synthesis of current knowledge on radon-related biomarkers.

To enhance methodological transparency and reproducibility in accordance with the PRISMA-ScR framework, a more detailed search protocol was employed. Searches were conducted in PubMed, Scopus, and Web of Science between 1990 and December 2025, restricted to peer-reviewed English-language studies. The representative query was as follows:

(“radon” [MeSH Terms] OR “radon” [All Fields]) AND (“biomarker” [All Fields] OR “biological marker” [All Fields] OR “molecular marker” [All Fields]) AND (“DNA damage” [All Fields] OR “epigenetic” [All Fields] OR “microRNA” [All Fields] OR “lung cancer” [All Fields])

**Figure 1:**
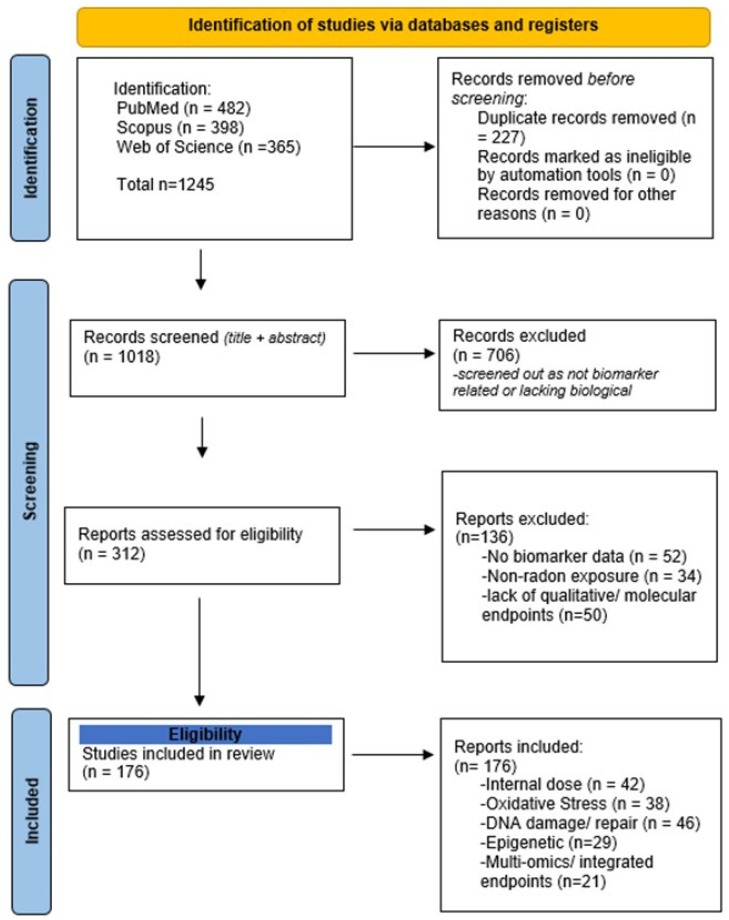
PRISMA flow diagram illustrating the identification, screening, and inclusion process for screening the scoping review. Searches across PubMed (*n* = 482), Scopus (*n* = 398), and Web of Science (*n* = 365) yielded a total of 1245 records. After removal of duplicates of 227 duplicate records, 1018 unique titles and abstracts were screened. Of these, 706 records were excluded for not meeting the inclusion criteria (e.g., studies unrelated to biomarkers or radon exposure). A total of 312 full-text articles were assessed for eligibility, and 136 studies were excluded for the following reasons: no biomarker data (*n* = 52), non-radon exposure (*n* = 34), or lack of quantitative or molecular endpoints (*n* = 50). Finally, 176 studies were included in the qualitative synthesis, distributed across biomarker categories as follows: internal dose (*n* = 42), oxidative stress (*n* = 38), DNA damage/repair (*n* = 46), epigenetic biomarker including microRNA (*n* = 29), and multi-omics or integrated endpoints (*n* = 21). A completed PRISMA-ScR checklist (Supplementary Checklist S1) accompanies this figure to verify compliance with reporting standards.

Equivalent Boolean logic was adapted for Scopus and Web of Science searches. The database search yielded 1245 records, of which 227 duplicates were removed, leaving 1018 unique records for title and abstract screening. 312 full-text articles were subsequently assessed for eligibility, and 176 studies ultimately met the inclusion criteria. Screening was performed independently by both authors, and disagreements were resolved by discussion. Full-text exclusions (*n* = 136) were due mainly to the absence of biomarker data (*n* = 52), non-radon exposure focus (*n* = 34), or lack of quantitative or molecular endpoints (*n* = 50). These quantitative details were also summarized in the updated PRISMA flow diagram (Figure 1) and further supported by the completed PRISMA-ScR checklist (Supplementary Checklist S1).

This review was conducted as a scoping narrative synthesis following the PRISMA-ScR guidelines [27] (Figure 1) to ensure transparent reporting of literature identification, screening, and inclusion. The accompanying PRISMA-SCR checklist (Supplementary Checklist S1) documents compliance with all 22 reporting items, including rationale, eligibility criteria, data charting process, and synthesis of results, ensuring full transparency of the literature selection process.

## 3. Results and Discussion

### 3.1. Biomarker Classification

**Figure 2:**
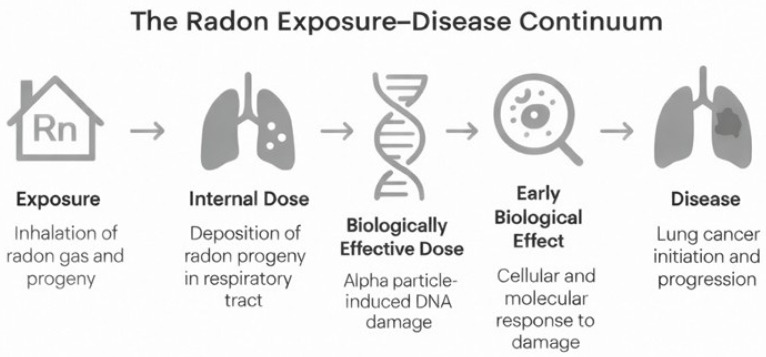
Radon exposure–disease continuum illustrating the step-by-step progression from environmental exposure to radon gas and progeny through internal deposition, biological effective DNA damage, early molecular changes, and eventual development of cancer.

Radon exposure leads to biological effects through a clearly defined pathway, starting from environmental contact and, in some cases, progressing to malignant transformation of lung tissues. Biomarkers, measurable indicators of biological processes, are vital for tracking this trajectory. To manage this complexity, this paper uses a classification framework based on definitions from the National Academy of Sciences (NAS), endorsed by the WHO, which categorizes biomarkers into three main functions: markers of internal dose, biologically effective dose, and early biological effect. Figure 2 illustrates a conceptual step-by-step overview of the complex cascade linking radon exposure to lung carcinogenesis.

#### 3.1.1. Rationale for Biomarker Classification Frameworks

Several frameworks for organizing biomarkers of environmental exposure exist. However, the model used in this review—the National Academy of Sciences (NAS) and World Health Organization (WHO) tripartite classification—most closely aligns with the biological processes specific to ionizing radiation. Other systems, like the exposure–effect–susceptibility (EES) framework often employed in chemical toxicology, focus on absorption, metabolic changes, and individual susceptibility [28]. While effective for metabolic agents, this model does not adequately represent the random, localized energy deposition typical of alpha-particle interactions resulting from radon decay. The NAS/WHO three-tier framework categorizes biomarkers into internal dose, biologically effective dose, and early biological effects, aligning with the sequence of radon-induced tissue damage. Internal dose biomarkers (such as ^210^Po and ^210^Pb) indicate radionuclide retention and decay within the body [29]. Biomarkers of biologically effective dose (like γ-H2AX, 53BP1 foci, and micronuclei) show direct DNA and chromosomal damage caused by alpha particles [30]. Early biological effect markers (such as 8-oxo-dG, changes in DNA methylation, and miR-21 dysregulation) reveal ongoing molecular and functional alterations linked to cellular transformation [31]. This structure offers both conceptual clarity and practical usefulness, enabling biomarkers to be placed along a continuous exposure–effect spectrum. By connecting physical dosimetry with molecular and cellular responses, the NAS/WHO framework provides a biologically consistent foundation for integrating diverse biomarker data across the radon research field.

#### 3.1.2. Classification of Radon-Related Biomarkers

Biomarkers of internal dose indicate the presence or burden of radon progeny within the body. Unlike environmental measurements like ambient radon gas levels (usually shown in Bq/m^3^), internal dose biomarkers reflect how much radioactive material has actually entered and remained in biological systems. For radon, these include radionuclides such as polonium-210 (^210^Po) and lead-210 (^210^Pb), which can be detected in bones, teeth, or soft tissues. These decay products serve as integrators of exposure over months or years and can offer valuable retrospective data, especially in occupational groups where historical environmental data might be incomplete. These internal metrics are essential because they inherently capture an individual’s personal exposure history, including inhalation processes, aerosol deposition, and physiological clearance. Unlike air monitoring, which varies over time and space, internal biomarkers provide a biologically integrated measure of cumulative exposure. This approach has been highlighted in population studies and animal models using ^210^Pb as a long-term dosimetric marker [32].

More relevant to biological risk are biomarkers of biologically effective dose, which reflect molecular events triggered by alpha particles interacting with critical cellular targets, mainly DNA. High-LET alpha particles from radon progeny like ^218^Po and ^214^Po deposit energy densely along a narrow tissue path, causing clustered DNA damage that is difficult for cells to repair precisely. These biomarkers include cytogenetic changes such as chromosomal aberrations (e.g., dicentrics and translocations), micronuclei formation, and direct detection of DNA double-strand breaks through γ-H2AX foci. Additional methods like the comet assay can identify strand breaks and alkali-labile sites at the single-cell level. These endpoints not only verify that exposure took place but also show that energy was deposited into biologically sensitive areas, particularly basal and secretory epithelial cells in the bronchial lining, known targets in radon-related cancer development [22]. Since these biomarkers often correlate with exposure levels (e.g., in working level months), they are especially useful in epidemiological research. A thorough understanding of how these biomarkers differ from those indicating just radionuclide retention is essential for interpreting biological responses to radon exposure. Table 1 compares the main features of internal dose biomarkers and biologically effective dose biomarkers, emphasizing their sample types, temporal sensitivity, specificity, and analytical techniques. This comparison highlights how each provides unique yet complementary insights along the radon exposure–response continuum.

**Figure 3:**
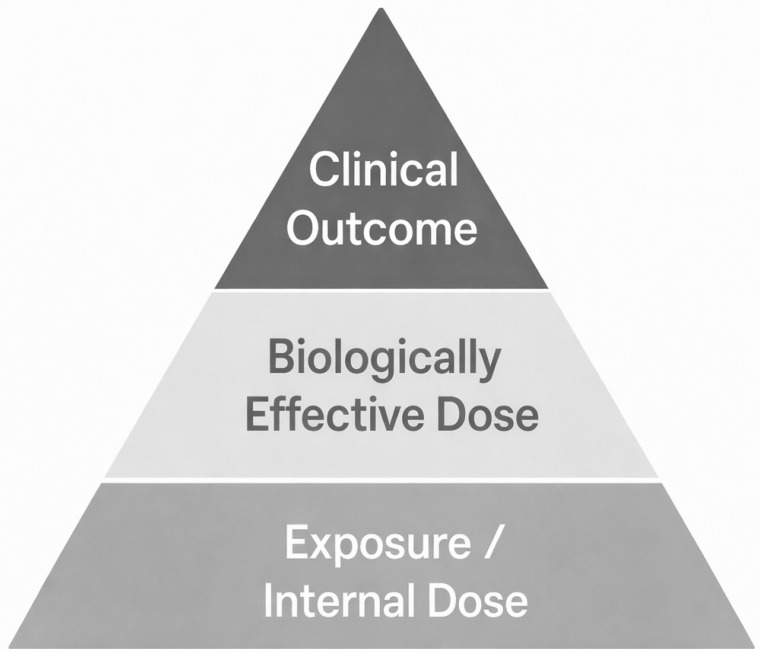
Progression of biomarker development along the radon exposure and disease axis. The base represents environmental exposure and internal dose biomarkers that quantify absorbed radionuclides, advancing upward through biologically effective dose markers that detect molecular damage. This leads to early biological effect biomarkers reflecting cellular and functional responses, culminating at the top with clinical manifestation.

Biomarkers indicating early biological effects mark an important next step in understanding exposure-related disease processes. These markers highlight cellular and molecular responses resulting from ongoing or unrepaired damage, serving as signs of either repair efforts or progression toward cancer. In the case of radon exposure, early indicators include somatic mutations in key cancer genes such as TP53 and KRAS, epigenetic changes like DNA methylation and histone modifications, microRNA expression shifts, and increased activity of genes related to inflammation and oxidative stress. Proteomic markers, including elevated cytokine levels and autoantibodies targeting tumor-associated antigens, are also valuable. These early changes can be detected in accessible biological samples like blood or sputum, enabling non-invasive monitoring. Their prognostic significance lies in reflecting a biological response that has advanced from DNA damage to functional change. When combined with internal and effective dose biomarkers, these signatures deepen understanding of disease progression at a molecular level [36]. This progression from molecular damage to functional alteration can be seen as a hierarchical continuum of biomarker development, shown in Figure 3. Each level, from exposure and internal dose at the bottom to biologically effective damage, early biological effects, and finally clinical disease at the top, represents a successive stage in the development of radon-induced lung cancer.

Overall, this classification system offers a scientifically consistent and practical model for discovering and applying biomarkers in radon research. It enables researchers to precisely place each biomarker within a causality framework and choose suitable endpoints for exposure tracking, mechanistic investigations, or early detection. This structure also supports translational aims: biomarkers of internal and biologically effective doses help improve individual risk assessments, while early effect biomarkers show potential for finding pre-symptomatic individuals at elevated risk of lung cancer.

The following sections provide a detailed discussion of these three biomarker categories. Section 3.2 examines markers related to internal dose, such as radionuclide buildup and biokinetic models. Section 3.3 focuses on DNA damage and cytogenetic markers as indicators of biologically effective dose. Section 3.4 analyzes molecular and immunological signatures associated with early biological effects. This structured approach offers a thorough framework for improving biomarker-guided radon risk assessment and early intervention strategies.

#### 3.1.3. Integrated Mechanistic Framework for Radon-Induced Biomarker Pathways

**Figure 4:**
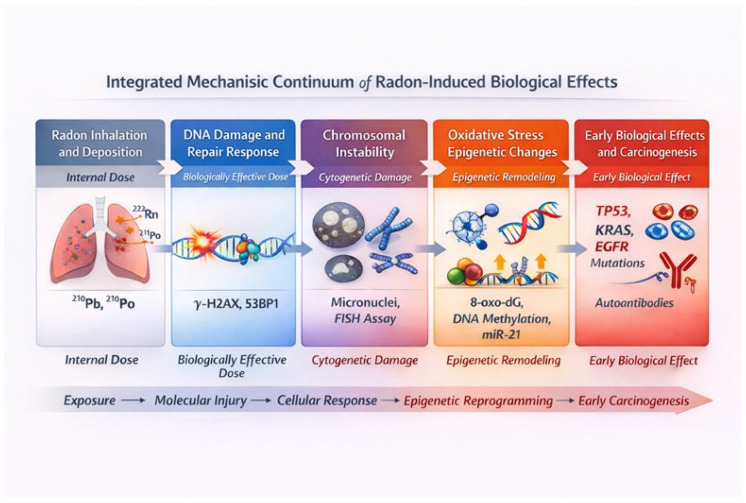
Integrated mechanistic continuum of radon-induced biological effects. The sequential pathway shows how radon exposure leads from DNA damage and chromosomal instability to oxidative, epigenetic changes that drive early lung carcinogenesis.

To better connect the different biomarker classes within a single biological narrative, this paper proposes an integrated mechanistic framework that links environmental radon exposure to downstream molecular and cellular events leading to disease, as illustrated in Figure 4.

The process starts with the inhalation and deposition of radon progeny, mainly ^218^Po and ^214^Po, whose α-particles deliver high-linear energy transfer radiation to the bronchial epithelium. This causes clustered DNA damage, including double-strand breaks, base lesions, and DNA-protein crosslinks, all concentrated within a few micrometers of tissue. These initial damages trigger the DNA damage response (DDR) pathway, characterized by rapid phosphorylation of histone H2AX and recruitment of 53BP1 proteins. The formation of *γ*-H2AX/53BP1 foci serves as a measurable biomarker of biologically effective dose and is the earliest molecular evidence of *α*-particle interaction with nuclear DNA. When repair is incomplete or prone to errors, misrepaired DNA lesions can lead to chromosomal aberrations and micronuclei. These changes may persist over time and can be detected through fluorescence in situ hybridization (FISH) or the cytokinesis-block micronucleus assay (CBMN). Such cytogenetic alterations serve as direct indicators of accumulated genotoxic stress.

Prolonged or repeated exposure leads to oxidative stress becoming a primary injury mechanism. Radon-induced radiolysis of water and mitochondrial dysfunction produce reactive oxygen species, which oxidize nucleic acids and lipids, generating biomarkers like 8-oxo-2′-deoxyguanosine (8-oxo-dG) and malondialdehyde (MDA). Ongoing oxidative and inflammatory signals drive epigenetic changes, such as global DNA hypomethylation, histone modifications at specific promoters, and microRNA dysregulation, including miR-21 and miR-34a. These alterations create a semi-permanent “epigenetic memory” of exposure that can remain even after environmental radon levels decline. The final stage involves the molecular fixation of damage and early malignant changes, marked by somatic mutations in TP53, KRAS, and EGFR, along with dysregulated oxidative and inflammatory gene networks and the development of autoantibody responses to tumor-associated antigens. Collectively, these events create a mechanistic continuum, with each biomarker class (internal dose, biologically effective dose, and early biological effect) representing a distinct yet interconnected phase of the radon exposure–disease pathway. This integrative framework emphasizes that biomarkers should not be viewed as isolated endpoints but as interconnected indicators of a continuous biological process. Recognizing the progression from physical exposure to molecular and systemic outcomes offers a foundation for multi-omic approaches that can connect environmental dose with personalized biological risk measures.

### 3.2. Internal Dose

The accurate quantification of internal dose biomarkers is central to mechanistic and epidemiological studies on Radon-222 (^222^Rn)-induced lung cancer. While ambient radon gas measures (in Bq/m^3^) provide an estimate of environmental concentration, biomarkers of internal dose capture the actual uptake, retention, and biological persistence of radon progeny within human tissues, thereby offering a more direct link to radiobiologically relevant exposure.

#### 3.2.1. Direct Measurement of Environmental and Biological Radon Progeny

Radon gas itself is inert and is mostly exhaled. The primary health risk stems from its short-lived solid progeny, which deposits in the respiratory tract, decays into longer-lived isotopes, and can accumulate in bones, teeth, or other tissues. Among these, lead-210 (^210^Pb), with a physical half-life of 22.3 years, and polonium-210 (^210^Po), with a half-life of 138 days, are the most widely used as internal dosimeter markers. For example, a 2025 study by Miller et al. [38] reported elevated toenail ^210^Pb/Pb isotope ratios in individuals with long-term high residential radon exposure (~545 Bq/m^3^ over 18.5 years), indicating a potential for non-invasive, personalized, retrospective dosimetry. Additional work involved directly measuring the activity of ^210^Po implanted on glass surfaces to reconstruct past exposure and link it to indoor radon concentrations [39]. The use of calcified tissues like bone or teeth is based on their capacity to accumulate and hold radionuclides over extended periods, functioning as records of total exposure. A 2021 study found that ^210^Po measured in vivo in skull bone using low-energy gamma spectrometry correlates reasonably well with long-term exposure, despite acknowledged uncertainties in background exposure and individual differences [40].

#### 3.2.2. Biokinetic Modeling and Retrospective Dosimetry

Direct measurement is essential; however, interpreting results relies on advanced modeling to convert radionuclide burdens into meaningful dose and exposure metrics. Established frameworks, such as the International Commission on Radiation Protection (ICRP) Publication 66 (which outlines the human respiratory tract model) and related biokinetic models, simulate the processes of deposition, translocation, retention, and clearance of inhaled radon progeny—including ^210^Pb and ^210^Po—across different organs. Recent research, for example, by Desorgher et al. [41], has utilized Monte Carlo micro-dosimetry techniques to estimate ^210^Po doses in rodent lungs, providing insights into organ-specific dose burdens. Meanwhile, residential retrospective dosimetry efforts include ^210^Po glass-implantation methods and correction models for equilibrium factors and aerosol behavior [39]. These integrated approaches enable estimation of cumulative radon progeny uptake in populations without historical measurements. Biokinetic modeling thus serves two main purposes: first, to convert measured tissue radionuclide burdens into estimates of lifetime radon progeny exposure or organ doses; second, to aid in retrospective reconstruction of epidemiologic studies, particularly for occupational cohorts like uranium or tin miners, where personal monitoring was not available or was historical.

#### 3.2.3. Advantages and Limitations of Internal Dosimetry Biomarkers

The strengths of internal biomarkers are significant. They naturally offer long-term exposure integration over months to decades, which helps smooth out fluctuations in radon levels and occupancy patterns. Additionally, they inherently include individual physiological factors such as breathing depth, aerosol deposition, and clearance that environmental radon measurements do not capture. For instance, the toenail ^210^Pb study by Miller et al. [38] highlights the greater usefulness of these biomarkers in assessing personalized exposure history. However, significant limitations remain. The low temporal resolution prevents biomarkers from distinguishing recent spikes from long-term chronic uptake, reducing their sensitivity to short-term changes in the radon environment or mitigation efforts. Accessing tissues like bone or teeth is challenging, often requiring invasive procedures, only being feasible post-mortem, or relying on surrogate tissues. Background sources of ^210^Pb/^210^Po from dietary and environmental sources can confound attributions solely to radon. Additionally, individual variability, measurement uncertainties, and assumptions within models of clearance and retention further complicate interpretation [40]. Nevertheless, when positioned at the base of the exposure–effect continuum, internal dose biomarkers serve as the fundamental quantitative substrate upon which biological effect markers are built. Because of their integrative nature, they remain essential anchors in mechanistic radon carcinogenesis models and retrospective epidemiologic reconstructions.

### 3.3. Biological Effective Dose

The concept of biologically effective dose sits at the intersection of physics and biology. It translates the energy from alpha particles emitted during radon decay into cellular and molecular damage that can lead to cancer. While biomarkers of internal dose measure the amount of radionuclides accumulated in the body, biomarkers of biologically effective dose reflect how much of that exposure has caused biological injury. These biomarkers serve as a mechanistic link between inhaled radon progeny and the start of cancer-related processes in the lung and other tissues. Because radon progeny emit high-LET (linear energy transfer) alpha particles, they deposit energy in highly localized spots, creating clusters of ionization within just a few micrometers. These concentrated energy tracks lead to dense, complex DNA damage that is much harder for the cell to repair compared to the diffuse damage from low-LET gamma or X-radiation. The biological markers of these events, such as chromosomal rearrangements, persistent DNA strand breaks, oxidative lesions, and dysregulated repair signals, serve as molecular fingerprints that biodosimetry aims to measure.

#### 3.3.1. Cytogenetic Biomarkers: Gold Standard in Radiation Biodosimetry

Cytogenetic assays remain the essential method for effective biodosimetry [42]. Chromosomes serve as a detailed record of radiation damage, and their aberrations (whether stable or unstable) are dependable indicators of the absorbed dose [43]. Unstable aberrations such as dicentrics, centric rings, and acentric fragments result from misrepaired DNA double-strand breaks (DSBs). These aberrations are easily identified in metaphase spreads of peripheral blood lymphocytes through the dicentric chromosome assay (DCA) [44]. This method has long been regarded as the gold standard for evaluating recent or acute exposures. Since these aberrations are transient and disappear as damaged lymphocytes are replaced, they are especially useful for assessing short-term or accidental exposures [45]. Numerous occupational and environmental studies, including those involving radiology staff and miners, have demonstrated a clear dose–response relationship between ionizing radiation exposure and the frequency of dicentric and ring chromosomes [46]. For reconstructing long-term or historical exposures, stable translocations identified through fluorescence in situ hybridization (FISH) provide more valuable information. Because these rearrangements endure through cell divisions, they reflect the cumulative, long-term biological impact [47,48]. Recent progress in FISH techniques now offers full-genome coverage. Advances in multicolor and multiplex FISH methods enable the detection of various aberration types in a single test, increasing both the precision and speed of dose estimation [49,50]. These stable aberrations have been found at higher frequencies even decades after occupational radon exposure, demonstrating their importance in retrospective biomonitoring. The cytokinesis-block micronucleus (CBMN) assay offers a practical, high-throughput alternative to analyzing metaphase cells. Micronuclei can originate from chromosomal breakage (clastogenic events) or entire chromosome loss (aneugenic events) during cell division, covering multiple damage pathways. Its simplicity and reproducibility have made it popular in biomonitoring populations exposed to radon over long periods, such as miners and residents in high-radon homes. Ongoing research confirms its sensitivity even at low doses typical of residential exposure [51,52]. Additionally, a 2023 multi-cohort study found consistent increases in micronuclei among healthcare workers exposed to radiation compared to those unexposed [53]. These cytogenetic assays provide complementary information over time: unstable aberrations signal recent exposure, whereas stable translocations and micronuclei suggest accumulated or long-term damage.

#### 3.3.2. DNA Damage and Repair Biomarkers

Advances in molecular dosimetry now allow for the visualization of DNA damage and repair processes at the single-cell level [54,55]. Alpha radiation’s limited range and high ionization density cause clustered double-strand breaks (DSBs), which are challenging to repair accurately [56]. These lesions activate the DNA damage response (DDR) network, coordinating repair by phosphorylating histone proteins and assembling repair complexes [57]. The phosphorylated histone variant H2AX quickly forms visible foci at DSB sites within minutes of exposure, serving as a highly sensitive indicator of recent radiation injury [58]. In radon inhalation experiments, γ-H2AX foci increase proportionally with the total radon dose, especially in lymphocytes and bronchial epithelial cells [59]. Nonetheless, since these foci are temporary and disappear within hours as DNA repair occurs, this test is most effective for detecting recent exposure or ongoing DNA damage rather than cumulative long-term effects [60]. The DNA repair protein 53BP1 (p53-binding protein 1) localizes with γ-H2AX at DSBs and serves as a scaffold for non-homologous end joining repair [58]. The colocalization of γ-H2AX and 53BP1 is a strong marker for the presence and processing of DSBs. A comparative study of peripheral blood lymphocytes irradiated with cobalt-60 gamma rays showed linear dose–response curves for both markers from 0.05 to 4 Gy, confirming their usefulness in human biodosimetry and triage after radiation exposure [61]. Notably, persistent colocalization of γ-H2AX and 53BP1 foci may indicate faulty repair mechanisms, which are linked to radiation sensitivity and increased cancer risk. The comet assay, also known as single-cell gel electrophoresis, remains a highly versatile method for identifying DNA strand breaks and alkali-labile sites [62]. When subjected to an electric field, damaged DNA fragments migrate from the nucleus, creating a comet-shaped tail whose length and brightness reflect the extent of damage. While it lacks the mechanistic detail of techniques like γ-H2AX or FISH, it effectively detects both single- and double-strand breaks across nearly all nucleated cells. Its affordability and low sample requirements have made it a popular choice for biomonitoring workers exposed to occupational hazards and populations affected by radon [63].

#### 3.3.3. Oxidative Stress and DNA Adduct Biomarkers

Beyond direct ionization, alpha radiation also damages DNA indirectly by radiolyzing water, which produces reactive oxygen species (ROS) like hydroxyl radicals and hydrogen peroxide. These ROS lead to oxidative damage of bases, strand breaks, and lipid peroxidation, resulting in continued genomic instability even after exposure [64,65]. Among oxidative lesions, 8-oxo-dG is the most extensively studied and is considered a reliable biomarker of oxidative stress [66,67]. Increased levels of urinary or serum 8-oxo-dG have been observed in radiation workers, miners, and patients receiving radiotherapy. Experimental research indicates that oxidative DNA damage frequently correlates with the rate of chromosomal aberrations, implying that ongoing oxidative stress plays a role in accumulating genotoxic damage [68]. When radon exposure happens in smokers or polluted environments, bulky DNA adducts from polycyclic aromatic hydrocarbons (PAHs) may amplify radon-induced damage [69]. Tobacco smoke increases ROS production and causes mutations in key genes like TP53 and KRAS, resulting in overlapping molecular signatures. This complicates epidemiological attribution but underscores the significant interaction between smoking and radon in the development of lung cancer [70]. These cytogenetic, molecular, and oxidative stress markers together define the biologically effective dose in the radon exposure–disease continuum. Unstable and stable chromosomal aberrations serve as physical traces of alpha-particle damage, while DDR biomarkers like γ-H2AX and 53BP1 reveal the molecular details of repair processes. Oxidative markers reflect the ongoing metabolic effects of radiation injury. When combined with internal dose biomarkers, they allow for a detailed reconstruction of both the exposure and its biological impact. This integrated, personalized dose–response approach improves risk assessment and helps identify individuals or groups with increased susceptibility to radon-induced lung cancer.

While various assays measure DNA damage from radon exposure, they each focus on different aspects of the damage repair process, and their responses may diverge under chronic low-dose conditions [71,72]. Combining these endpoints offers a more comprehensive understanding of radon’s biological effects. During acute or high-dose alpha-particle exposure, DNA double-strand breaks occur in clustered regions and are quickly detected as y-H2AX and 53BP1 foci, which peak within hours and usually resolve within 24–48 h if repaired successfully [58]. In contrast, chronic low-dose exposure causes persistent or misrepaired breaks that lead to stable chromosomal translocations and micronuclei formation. These abnormalities accumulate over weeks or months and act as long-term indicators of genomic instability [53,73]. The comet assay detects transient single- and double-strand breaks and oxidative base lesions, but it is highly sensitive to dose rate and repair capacity. During low-dose, prolonged exposure, comet tail moments may return to normal even if chromosomal aberrations remain. Conversely, fluorescence in situ hybridization (FISH) offers a stable record of accumulated translocations, showing a stronger correlation with long-term α-particle exposure than with short-term repair events [74,75]. Together, these endpoints demonstrate a hierarchical response: γ-H2AX and comet assays indicate immediate repair processes, while micronuclei and FISH-captured translocations reveal lingering or propagated damage typical of long-term low-dose exposures. Combining results from these tests can enhance biomarker accuracy and improve the understanding of timing in radon dosimetry. This comparison is summarized in Table 2, which outlines the key distinctions in sensitivity, repair kinetics, and interpretive value of major DNA-damage biomarkers across different exposure contexts.

### 3.4. Early Biological Effects

Biomarkers of early biological effects are the initial measurable cellular and molecular responses to radon exposure, occurring before clinical symptoms appear. They indicate the shift from an effective dose to visible cellular disruptions and reflect the combined effects of DNA damage, epigenetic changes, altered gene regulation, and immune system activation. In the context of radon-induced lung cancer, these biomarkers reveal the earliest stages of malignant transformation, bridging the gap between exposure and disease development.

#### 3.4.1. Mutation Signatures and Somatic Alterations

Alpha-particle irradiation creates a distinctive mutational fingerprint marked by small deletions, complex rearrangements, and clustered base substitutions, which reflect the densely ionizing nature of high linear energy transfer (LET) radiation [19,70,85]. Experimental systems utilizing the hypoxanthine-guanine phosphoribosyltransferase (HPRT) and thymidine kinase (TK) loci confirm their usefulness as mechanistic models for studying the mutagenic effects of radon progeny [86,87,88]. In human populations, particularly among uranium miners and individuals living in high-radon dwellings, somatic mutations in key cancer driver genes such as TP53, KRAS, and EGFR have been consistently observed. The TP53 tumor suppressor gene shows a pattern of A:T → C:G transversions and G → A transitions, aligning with the oxidative deamination and strand misrepair typical of alpha-radiation damage [70,89]. Mutations in the KRAS oncogene, especially at codon 12, have been reported in lung tumors from radon-exposed miners [90]. Additionally, alterations in EGFR, ALK, and ROS1 are increasingly identified among non-smoking lung cancer patients in radon-prone areas [91]. These molecular features, seen in non-small-cell lung carcinoma (NSCLC), sometimes overlap with those caused by tobacco carcinogens, but in many cases, are uniquely linked to the biophysical effects of alpha radiation. Next-generation sequencing (NGS) has significantly enhanced the ability to detect early mutational events. By applying deep sequencing to lung tissue and plasma DNA, researchers have identified clonal expansions containing TP53 and KRAS mutations in the histologically normal bronchial epithelium of individuals exposed to radon [92]. These results suggest that radon initiates mutational seeding within field epithelial cells, from which malignant clones can develop. Complementary work in murine models indicates that such early clones can persist without forming overt tumors, reinforcing the idea that mutational persistence may represent a latent carcinogenic risk phase. Additionally, integrated genomic analysis shows that co-occurring TP53 and KRAS mutations characterize particularly aggressive lung adenocarcinoma subtypes, driven by dysregulated RNA methylation and overexpression of cyclin B1 [93]. These findings demonstrate that radon’s genotoxic effects are not only mutagenic but also selectively influence clonal trajectories toward malignancy.

#### 3.4.2. Epigenetic Modifications

While modifications create a permanent record of DNA damage, epigenetic changes represent a more dynamic yet equally significant aspect of radon’s biological effects. Ionizing radiation alters the epigenome through lasting changes in DNA methylation, histone architecture, and non-coding RNA networks, leading to reprogrammed cellular identity and gene expression. Genome-wide methylation studies on lung tissues and blood samples from radon-exposed individuals reveal a consistent dual signature: global DNA hypomethylation, which destabilizes repetitive elements and promotes chromosomal instability, and promoter-specific hypermethylation of tumor suppressor genes such as CDKN2A, RASSF1A, and MGMT, leading to their functional silencing [94]. These changes correlate with increasing exposure and persist long after exposure ends, suggesting they serve as an epigenetic memory of radiation exposure. Independent validation through genome-scale methylation analysis in occupational cohorts supports these findings, highlighting dose-dependent CpG island hypermethylation in DNA repair and cell-cycle genes [95]. Alpha radiation significantly impacts histone post-translational modifications. Studies have shown decreases in histone H4 acetylation and changes in H3K9 and H3K27 methylation patterns after long-term radon exposure in vitro. These findings suggest that chromatin condensation and limited DNA accessibility lead to reduced DNA repair accuracy [89]. This ongoing repressive chromatin state promotes genomic instability and supports the idea of radiation-induced epigenetic inheritance, where even non-irradiated progeny cells maintain altered transcription profiles. Among the most promising epigenetic biomarkers are circulating microRNAs (miRNAs), small non-coding RNAs that regulate gene expression after transcription [96]. miRNAs such as miR-21, miR-34a, and miR-222 are upregulated in response to alpha radiation and play key roles in apoptosis, DNA damage signaling, and epithelial–mesenchymal transition [97]. Elevated plasma miR-21 levels have been observed in both radon-exposed workers and lung cancer patients, where they show an inverse relationship with the tumor suppressor PTEN expression [98]. Because they are stable in blood and easy to measure, circulating miRNAs present a promising option for non-invasive biomonitoring and early detection of disease in populations exposed to radon.

#### 3.4.3. Gene Expression Alterations

The transformation from DNA and epigenetic damage to changes in gene expression represents another important biomarker area. Transcriptomic profiling of blood and lung cells from individuals exposed to radon consistently shows increased expression of genes involved in DNA damage response, apoptosis, inflammation, and oxidative metabolism [99]. These findings suggest that radon exposure triggers a coordinated network of stress-response pathways [100]. However, prolonged activation of these pathways might increase the risk of malignant transformation in tissues. Recent transcriptomic studies have shown that peripheral blood mononuclear cells from miners exposed to radon exhibit significant upregulation of genes like GADD45A, ATM, and FAS, along with inflammatory cytokines such as IL-6 and IL-8 [70]. This gene expression pattern resembles that seen in irradiated lung tissue, indicating systemic responses can be identified even in accessible peripheral samples [101]. The persistent activation of antioxidant defense genes (SOD2, GPX1, NQO1) suggests ongoing oxidative stress, likely due to both direct reactive oxygen species (ROS) production and secondary effects of chronic inflammation [102]. Collectively, these transcriptional changes outline a molecular profile of cells caught in a cycle of damage detection and incomplete repair, a characteristic feature of premalignant stress adaptation.

#### 3.4.4. Proteomic Biomarkers and Autoantibodies

At the protein level, radon exposure induces complex responses involving inflammation, tissue remodeling, and immune recognition of damaged self-proteins. Proteomic studies of plasma and bronchoalveolar lavage fluid have shown increased levels of cytokines like interleukin-6 (IL-6), interleukin-8 (IL-8), and tumor necrosis factor-α (TNF-α), as well as growth factors such as vascular endothelial growth factor (VEGF) and epidermal growth factor (EGF). Elevated oxidative stress markers, including carbonylated proteins and peroxiredoxins, support these findings, indicating an ongoing redox imbalance and immune activation [70]. An intriguing advance in early detection involves the discovery of autoantibodies targeting tumor-associated antigens (TAAs). These autoantibodies develop as the immune system reacts to abnormal or misfolded proteins produced by early cancerous cells. In lung adenocarcinoma, autoantibodies against p53, cytokeratin fragments, and centromere proteins such as CENPF have been found in serum months prior to radiological or clinical diagnosis [103]. This suggests that immune surveillance is activated early in radon-induced carcinogenesis and that these autoantibody profiles could serve as predictive biomarkers for early disease progression. Combining immunoproteomic signatures with genomic and epigenetic data may improve the sensitivity of screening methods for radon-related lung cancer. The combined insights from genetic, transcriptomic, and proteomic studies form a clear narrative: Radon exposure triggers complex biological responses that progress from local DNA damage to widespread molecular dysregulation. Mutations in key oncogenes and tumor suppressors create conditions for cellular transformation, while epigenetic changes and ongoing oxidative stress lock these alterations into abnormal but stable cellular programs. Changes in gene expression and proteomics then translate these internal disruptions into detectable systemic signals, some of which can be measured without invasive methods. This integrated perspective redefines early biological effect biomarkers, viewing them not just as passive indicators of exposure but as active players in lung cancer development. When used within multi-omics frameworks, these biomarkers offer a strong basis for precise risk assessment, early detection, and prevention in populations exposed to radon over time.

In summary, biological effects offer the clearest molecular evidence of the transition from exposure to disease initiation in radon-induced lung cancer. Mutational signatures in TP53 and KRAS, circulating miRNAs such as miR-21, and autoantibodies against tumor-associated antigens emerge as promising early indicators with translational potential for screening and risk assessment. These biomarkers collectively form a mechanistic continuum linking DNA damage, epigenetic dysregulation, and immune activation, laying the groundwork for future biomarker panels that combine sensitivity with clinical practicality. Many molecular changes resemble those caused by general ionizing radiation, but radon progeny create uniquely localized, high linear energy transfer (LET) alpha-particle tracks that produce dense ionization clusters within tissue regions on a micrometer scale. These high-LET interactions generate complex DNA damage and chromosomal rearrangements that are repaired less efficiently than low-LET damage from X-rays or gamma rays. Additionally, characteristic mutational patterns such as TP53 A:T→C:G transversions and KRAS codon 12 substitutions, along with ongoing oxidative stress and inflammatory signals, serve as key molecular markers of radon exposure. Identifying these specific signatures is crucial for differentiating radon’s carcinogenic process from other effects of ionizing radiation.

### 3.5. Methodological Challenges in Biomarker Application

Although there have been major advances in identifying biomarkers that indicate radon exposure and biological effects, turning these molecular signals into reliable epidemiological tools is still challenging. Variations in exposure levels, genetic backgrounds, lifestyle factors, and methodological inconsistencies often make it difficult to establish clear dose–response relationships, especially in populations exposed to low-level or chronic radiation. Therefore, a careful assessment of the methodological limitations related to biomarker use is crucial to understanding their validity in both population-based and occupational research.

#### 3.5.1. Sensitivity and Specificity for Low-Level, Chronic Exposure

A key challenge in biomarker applications is the limited sensitivity of current assays to detect the biological effects of low-level, long-term radon exposure [104]. Unlike acute radiation incidents, where cytogenetic and molecular markers clearly show a dose–response, chronic exposures produce subtler effects that often fall below the detection capabilities of present analytical techniques [85]. While biomarkers like γ-H2AX foci and micronucleus frequencies are responsive to short-term exposure peaks, they may underestimate the overall biological burden when radiation occurs at low doses over extended periods [58,59]. Additionally, biological repair and adaptation mechanisms can diminish or conceal these effects, leading to non-linear or threshold-like responses that complicate risk assessment in the general population [85,105].

#### 3.5.2. Key Confounders: Tobacco, Diet, Age, and Co-Exposures

The interpretation of biomarker data is further complicated by strong confounding variables. Tobacco smoking remains the most significant factor, acting both synergistically and as a competing source of oxidative DNA damage [106]. The combination of radon damage and tobacco exposure leads to a supra-additive risk for lung cancer due to overlapping mechanisms such as oxidative stress and DNA adduct formation [107]. Additionally, tobacco smoke influences DNA repair gene expression, alters DNA methylation patterns, and increases baseline chromosomal aberrations, all of which can inflate biomarker readings independently of radon exposure [108,109]. Age is another key factor affecting biomarker variability [110]. As cells age, they accumulate spontaneous chromosomal translocations and oxidative damage, which makes it challenging to differentiate between natural molecular noise and effects caused by radiation [111]. Factors like dietary antioxidants, alcohol consumption, and exposure to environmental carcinogens such as arsenic and silica further influence oxidative balance and DNA repair, increasing variability among individuals [112]. This interplay highlights the importance of careful adjustment and stratification in biomarker-based epidemiological studies.

#### 3.5.3. Genetic Polymorphisms in DNA Repair (XRCC1, OGG1, XRCC3)

Genetic variations in the DNA repair pathway are a key factor in individual susceptibility to radon-induced damage. Polymorphisms in base excision repair genes, particularly XRCC1 (Arg399Gln, Arg280His) and OGG1 (Ser326Cys), have been shown to affect the repair efficiency of oxidative and single-strand DNA breaks [113]. A recent meta-analysis involving 1648 radon-induced lung cancer cases found significant links between OGG1 (rs1052133), ERCC1 (rs3212986), and XRCC3 (rs861539) variants and increased cancer risk, while XRCC1 (rs25487) and ERCC2 (rs13181) did not show consistent associations [114]. Mechanistic studies further suggest that XRCC1 variants impact the stability and binding capacity of DNA ligase III within the repair complex, resulting in increased DNA strand breaks after alpha-particle exposure [115]. Likewise, OGG1 polymorphisms diminish the efficiency of excising 8-oxo-guanine, a key oxidative lesion, thereby exacerbating oxidative damage caused by radon and tobacco co-exposure [116]. These genetic variations help explain the significant interindividual differences in biomarker responses observed even among subjects with similar exposure levels, supporting the use of genotyping as a valuable tool in molecular epidemiology.

#### 3.5.4. Biological Variability: Inter- and Intra-Individual Differences

Biological variability remains a consistent challenge in interpreting biomarkers. Interindividual differences stem from variations in metabolism, immune status, hormonal environment, and baseline inflammatory levels. Conversely, intra-individual variability reflects the temporal dynamics of biomarker expression. For example, γ-H2AX and 53bp1 foci form within minutes of exposure but decline rapidly as DNA repair advances, while stable chromosomal translocations can persist for years. This temporal heterogeneity requires careful timing of sample collection in relation to exposure events to ensure accurate comparisons [58]. Additionally, fluctuating environmental conditions, seasonal variations, circadian effects on DNA repair gene expression, and sample handling differences introduce further sources of error that may overshadow true exposure effects.

#### 3.5.5. Tissue Source Considerations: Peripheral Blood vs. Target Tissue

A significant practical limitation in human biomarker research involves selecting the appropriate biological material. Many studies use peripheral blood lymphocytes (PBLs) as a surrogate tissue to evaluate genotoxic and epigenetic changes. While easily accessible, PBLs may not accurately reflect the molecular environment of the bronchial epithelium, which is the main site of radon-related carcinogenesis [117]. DNA damage and repair responses in PBLs might vary in extent or timing compared to lung epithelial cells, especially concerning oxidative stress and chromatin modifications. Techniques like bronchial lavage, sputum cytology, or exhaled breath condensates offer better insights into the pulmonary microenvironment but are challenging to implement for large-scale screening due to logistical and ethical concerns. As Vocht et al. showed, peripheral biomarkers such as DNA methylation can still reveal systemic effects of radon exposure, although tissue-specific responses are generally more indicative of localized disease processes [118].

#### 3.5.6. Design Challenges in Epidemiological Studies

Epidemiological studies that aim to incorporate biomarkers into exposure–response frameworks encounter various design limitations. Exposure misclassification is a widespread issue, especially when using short-term radon measurements as proxies for long-term exposure. Biomarker instability also adds difficulty to retrospective analyses, since some molecular endpoints tend to degrade or normalize over time [96]. Additionally, statistical power is frequently inadequate in small or diverse cohorts, especially when stratified by genotype or lifestyle factors [89]. Additionally, the complex relationship among exposure dose, repair capacity, and biomarker persistence necessitates longitudinal studies rather than cross-sectional snapshots [118]. Multilevel models that include genotypic, epigenetic, and lifestyle data show promise for addressing these interactions, but their effectiveness depends on standardized protocols and global data harmonization [119]. Currently, the absence of such standardization hampers inter-study comparison and meta-analyses. To clarify the interplay between biomarker interpretation and external modifiers, Table 3 provides a comparative overview of major biomarker classes, their primary confounding factors, and the specific ways these variables influence biomarker sensitivity and reliability in radon-exposed populations.

### 3.6. Biomarker in Human Studies

Human biomarker studies provide the empirical basis connecting radon exposure to biological effects and ultimately to disease risk. Over the last 60 years, biomarker research in radon epidemiology has evolved alongside advances in molecular biology, progressing from cytogenetic assays in miner cohorts to multi-omic analyses in residential populations. These applications have not only confirmed radon’s carcinogenic potential but also uncovered complex interactions among environmental dose, genetic susceptibility, and molecular responses.

#### 3.6.1. Occupational Exposure: Uranium, Tin, and Fluorspar Miners

Occupational cohorts have long been fundamental to radon epidemiology, offering the first solid dose–response evidence for lung cancer caused by radon progeny. The uranium miners on the Colorado Plateau, studied since the 1950s, established a causal relationship between cumulative radon exposure—measured in working level months (WLMs)—and lung cancer rates. Cytogenetic analyses in these miners consistently show increased frequencies of dicentric and ring chromosomes, micronuclei, and stable translocations, providing the earliest human evidence that alpha radiation causes a measurable genetic signature [120,121]. Further research on Wismut miners in Germany and Chinese tin miners expanded these findings, linking cumulative WLM exposure to increased chromosomal aberrations (Cas) and confirming fluorescence in situ hybridization (FISH) as the preferred method for detecting long-term cytogenetic damage. Studies of Wismut miners showed that stable chromosomal translocations identified by FISH remained detectable decades after exposure, even without ongoing contact with radon progeny [122,123]. Similarly, studies in Chinese cohorts found that miners exposed to over 100 WLM had notably higher rates of micronuclei and γ-H2AX foci compared to unexposed controls, with strong correlations to lung function decline and total dose [124,125]. Long-term follow-up of these occupational groups has been crucial in validating biometer persistence and dose reconstruction. For example, studies from the Uranium Miners Cohort in the Czech Republic showed that the rate of stable chromosomal translocations remained elevated over twenty years after exposure ended, highlighting their use as retrospective biodosimeters [126]. Overall, mining research has proven that cytogenetic biomarkers, together with epidemiological dose models, offer a robust method for quantifying biological effects in populations exposed to high radon levels.

#### 3.6.2. Residential Exposure: Global Case–Control and Cohort Studies

Despite the fact that miner cohorts established the dose–response paradigm, residential radon studies have aimed to adapt these findings to the general population, where exposures are typically lower and confounding factors are more complex. Landmark studies like the Iowa Radon Lung Cancer Study and the Swedish Residential Radon Study were among the first to use biomarker endpoints in non-occupational settings. The Iowa study combined long-term radon monitoring with assays for micronuclei and DNA damage in lymphocytes, revealing that even moderate residential exposure (≥150 Bq/m^3^) was linked to increased chromosomal aberrations and oxidative lesions [127].

European pooled analyses, especially those led by the European Radon and Lung Cancer Collaborative Group, have strengthened the statistical foundation of these findings. In a pooled review of more than 13 case–control studies involving 7000 lung cancer cases and 14,000 controls, a clear linear link was identified between residual radon exposure and lung cancer risk, even after adjusting for smoking and demographic factors [128]. Subsequent biomarker substudies have used measures like H2AX foci, comet assays, and epigenetic markers to assess early biological effects in residents. Recent research from Scandinavian and Central European cohorts shows consistent links between indoor radon levels and global DNA hypomethylation in blood cells, suggesting systemic oxidative stress responses [118].

Residential studies are increasingly adopting multi-omic methods, combining methylation, transcriptomics, and proteomics. For instance, a 2023 cohort study in Central Europe observed that epigenetic changes and increased inflammatory cytokines in peripheral blood closely correlated with indoor radon levels, even below 100 Bq/m^3^ [70]. This integration of molecular and environmental data strongly supports using biomarkers not just to measure exposure but also as predictive tools for early disease detection across the general population.

#### 3.6.3. Radon and Smoking Interactions: Challenges in Attribution

One of the most complex methodological and interpretive challenges in radon epidemiology is disentangling the synergistic relationship between radon exposure and tobacco smoking. Both factors cause DNA damage and mutations in overlapping genes, especially TP53 and KRAS, making it difficult to assign mutational patterns to one source [107]. Studies of lung tumors in miners and smokers have shown remarkably similar TP53 mutation hotspots, notably transitions at CpG dinucleotides, which reflect shared oxidative processes [129]. Epidemiological studies consistently support a model of risk where the combined effect of radon and smoking significantly exceeds the sum of their individual risks [130]. For example, data from North American and European miners show that the relative risk of lung cancer for smokers exposed to high radon levels can be more than 20 times higher, compared to about four times in non-smokers at similar exposures [131]. Mechanistically, tobacco smoke worsens radon-induced oxidative stress by depleting antioxidant defenses and hydrocarbon (PAH) adducts, which intensify radiation-related damage [100,108]. Molecular biomarker studies further indicate that smoking may alter the expression of radon-responsive genes and miRNAs, blurring molecular distinctions between the two exposures. For example, miRNA-21, a well-established onco-miR upregulated in both smokers and radon-exposed individuals, targets the PTEN and PDCD4 pathways, which are central to both inflammation and carcinogenesis [132,133,134]. These overlapping signatures make it difficult to identify radiation-specific biomarkers in populations exposed to mixed hazards [135], emphasizing the need for integrated biomarker panels capable of discriminating between synergistic and independent pathways of damage.

#### 3.6.4. Biomarker-Guided Risk Stratification

The ultimate goal of biomarker application in radon extends beyond exposure quantification toward risk stratification and early detection. Advances in genomics and proteomics have laid the groundwork for personalized assessment tools that could identify high-risk individuals within radon-exposed communities. Recent multi-omic models combining DNA methylation, miRNA expression, and inflammatory cytokine profiles have demonstrated strong predictive power for early lung tissue changes associated with radon exposure [70]. Integrating these molecular biomarkers with traditional exposure metrics (Bq/m^3^, WLM) enables a shift from population-based to individualized risk assessment, offering opportunities for targeted screening and intervention [136,137]. Liquid biopsy approaches, such as circulating cell-free DNA (cfDNA) sequencing and autoantibody profiling, have further proven their ability to detect subclinical molecular changes that precede tumor formation [138,139]. Biomarker-guided approaches could significantly transform radon mitigation efforts. By pinpointing individuals biologically more vulnerable—those with high biomarker levels despite only moderate environmental exposure—public health initiatives can focus screening and resource allocation more effectively while tracking remediation success over time. Additionally, ongoing biomarker monitoring in radon reduction trials can give real-time feedback on biological dose decreases, providing a clear indicator of public health improvements beyond just ambient air measurements. Table 4 summarizes the applications of biomarkers in both occupational and residential radon exposure studies.

### 3.7. Emerging Tools and Future Directions

Radon biomarker research is experiencing a transformative phase driven by advances in molecular biology, systems toxicology, and computational science. Although traditional cytogenetic and biochemical assays have established the foundation for exposure assessment, new technologies now provide unprecedented insights into how radon exposure disrupts biological systems across various molecular levels, including DNA, RNA, proteins, and metabolites. These emerging tools are redefining both the sensitivity and scope of biomarker discovery, facilitating a shift from population-level correlations to personalized molecular risk profiling.

#### 3.7.1. Mult-Omics Integration: Building Holistic Exposure Profiles

The multi-omics paradigm, combining genomics, epigenomics, transcriptomics, proteomics, and metabolomics, provides a comprehensive method to understand the biological response to radon exposure. Each omic layer offers a distinct view: genomics detects susceptibility variants in DNA repair and oxidative stress pathways (e.g., XRCC1, OGG1, TP53); epigenomics uncovers changes in methylation and histone modifications induced by exposure; proteomics measures dynamic signaling responses, while metabolomics monitors shifts in cellular redox state and energy metabolism. Recent integrative studies have demonstrated that omic data fusion significantly enhances exposure prediction accuracy and mechanistic understanding. For example, Kashkinbayev et al. [70] combined plasma proteomic and methylation data from radon-exposed individuals, identifying distinct inflammatory and metabolic signatures (elevated IL-6, TNF-alpha, and altered SOD2 methylation) that correlated with lung tissue injury markers. Similarly, Huang et al. [161] employed integrative transcriptomic and metabolomic profiling in bronchial epithelial cells, revealing perturbations in glycolysis and glutathione metabolism pathways consistent with oxidative stress from alpha-particle exposure. Multi-omics analysis offers a systems-level approach to identify biomarker panels that indicate both exposure level and biological effects. This provides a comprehensive, time-sensitive view of how radon influences cellular function.

However, implementing multi-omics approaches in radon research presents notable methodological and logistical challenges. High-dimensional omics data are highly susceptible to batch effects caused by differences in sample collection, sequencing platforms, and analytical pipelines, which can obscure true exposure-related signals [162,163]. The absence of standardized bioinformatics workflows further complicates cross-study comparisons, as variations in normalization, integration algorithms, and statistical modeling can result in inconsistent biological interpretations [164]. Additionally, comprehensive multi-omics profiling is resource-intensive, requiring large sample volumes, high sequencing depth, and advanced computational infrastructure, which are often difficult to access [165]. To sustain population-based or occupational studies, addressing these challenges requires coordinated efforts in data harmonization, cross-laboratory calibration, and developing shared reference datasets to guarantee reproducibility and scalability. Recognizing these constraints offers a balanced view of the potential of multi-omics approaches, highlighting that although they have transformative possibilities, their application in large radon-exposed populations will rely on systematic standardization and cost-effective analytical solutions. In the future, standardized multi-omic data across different cohorts will be essential for developing predictive models that combine environmental exposure, host genetics, and molecular phenotypes. Metabolomic biomarkers face unique reproducibility challenges compared to pre-analytical variables like sampling, timing, fasting, circadian rhythms, storage temperature, and physiological stress. Without strict standard operating procedures (SOPs) for sample handling and data normalization, the comparability between studies and the ability to reproduce results quantitatively are limited [166,167,168]. These issues also hinder regulatory acceptance and make metabolomics harder to translate into field applications compared to DNA-based markers. Recognizing these challenges highlights the importance of rigorous pre-analytical standardization to enable reliable integration of metabolomic assays into radio biomarker frameworks. Despite its powerful mechanistic capabilities, integrating multi-omics can lead to what is called “omics overreach”, where the effort to gain detailed biological insights surpasses what is practical for population or occupational settings. To translate these findings effectively, it is necessary to select a smaller set of omics-based signatures into simple, reliable biomarker panels that maintain strong predictive power while being feasible to analyze [169]. Striking a balance between scientific depth and logistical practicality is crucial to ensure that multi-omics methods genuinely contribute to, rather than impede, real-world radon risk assessment and monitoring.

#### 3.7.2. Single-Cell Approaches to Understanding Tissue Heterogeneity

A major limitation of bulk biomarker analysis is its failure to distinguish the cellular heterogeneity in the lung’s response to alpha-particle radiation [19,112]. Radon progeny mainly deposit in the bronchial epithelium, but the biological responses vary widely. Basal, secretory, and club cells show significantly different patterns of DNA repair, oxidative stress response, and apoptosis [20,170]. Single-cell omics techniques, such as scRNA-seq and scATAC-seq, have transformed radiation biology by uncovering cell-type-specific transcriptional and chromatin changes. Recent research with scRNA-seq indicates that radiation exposure leads to the emergence of distinct lung epithelial subpopulations, including progenitor-like basal cells that activate pathways involving TP53, GADD45A, and NFE2L2, which are associated with DNA repair and managing oxidative stress [161,171,172]. Similarly, single-cell chromatin profiling (scATAC-seq) has uncovered remodeling of enhancer regions associated with cell-cycle control and immune regulation triggered by exposure [173,174]. This provides molecular insights into how radon may contribute to early field cancerization. By integrating these datasets, researchers can trace lineage-specific injury and repair pathways, revealing how certain epithelial subpopulations either sustain or evade genotoxic damage. Despite these advances, applying single-cell and lineage-tracing technologies to populations exposed to radon remains both technically and logistically challenging. Generating high-quality single-cell datasets requires fresh tissue or carefully preserved biospecimens, which are often unavailable in large-scale epidemiological or occupational studies. These methods are also resource-intensive, needing deep sequencing coverage, advanced computational infrastructure, and specialized bioinformatics expertise. Additionally, batch effects and platform variability can mask subtle biological differences between exposure groups. The lack of standardized analytical pipelines further limits reproducibility across labs. To enable reliable and scalable use, future research should focus on protocol standardization, shared reference cell atlases, and cross-laboratory calibration to ensure consistent interpretation of single-cell data in radiation biology. Applying these findings to human bronchial organoids and ex vivo lung models is a vital next step, helping to connect molecular profiling with actual disease processes.

#### 3.7.3. Organ-on-a-Chip and 3D Lung Models

Organ-on-a-chip and 3D lung organoid technologies are gaining importance for studying how lung tissue responds to ionizing radiation. For example, a lung alveolus-on-a-chip lined with human alveolar epithelium and pulmonary endothelium recently demonstrated radiation-induced DNA damage, cytokine release, and barrier dysfunction in vitro [175]. Although application-specific data on alpha-particle or radon progeny exposure are limited, current platforms that replicate human lung structure, airflow, and cellular interactions show significant potential for examining the localized high-LET damage caused by radon progeny decay. Complementary studies using microfluidic pulmonary microvascular models have identified proteins and cytokine biomarkers that respond to radiation, emphasizing their usefulness for real-time mechanistic research on radon toxicity [176]. Similarly, human airway epithelial organoids have demonstrated lasting impairment of progenitor cell function following ionizing radiation, highlighting their importance for studying long-term tissue remodeling and repair processes [177]. Lung organoids created from human pluripotent stem cells further enable the modeling of chronic, fractionated exposures and demonstrate ongoing activation of inflammatory and epigenetic pathways [178].

#### 3.7.4. Practical and Translational Feasibility for Major Biomarker Classes

While the previous section outlined emerging technologies, the effective incorporation of radon-related biomarkers into population or occupational monitoring relies on their analytical feasibility, scalability, and infrastructure needs. Table 5 summarizes the primary biomarker classes discussed in the review and compares them based on measurement platforms, throughput, cost, and practicality.

As shown in Table 5, immunoassays and cytogenetic-based methods are still the most practical for field deployment due to their low cost and higher throughput, while omics-level assays are currently limited by high analytical costs and the need for specialized infrastructure. Understanding these practical differences can help prioritize biomarkers with the greatest near-term potential for inclusion in radon monitoring frameworks.

#### 3.7.5. Decision-Oriented Synthesis of Radon Biomarkers

To offer clearer transitional guidance, Table 6 summarizes the current maturity of biomarker classes in radon research. Biomarkers are ranked based on biological relevance, technical feasibility, and translational readiness, emphasizing both near-term opportunities and exploratory frontiers.

**Figure 5:**
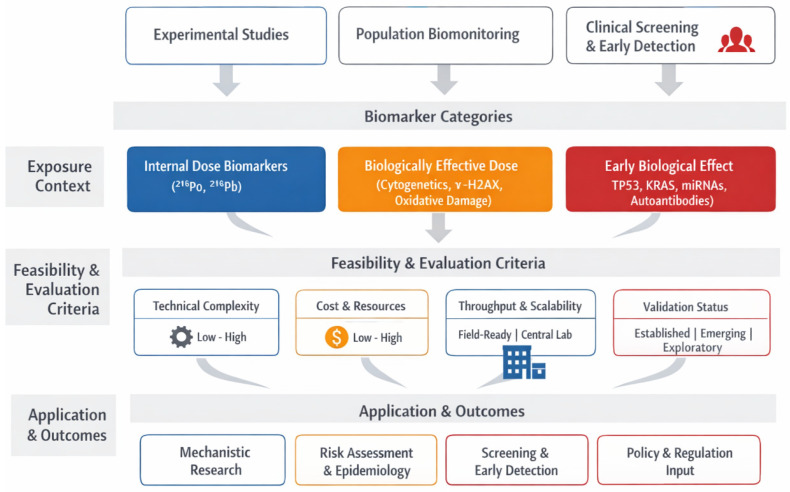
Operational decision pathway for selecting and applying radon biomarkers. The schematic outlines a tiered approach linking biomarker class, feasibility and application level (from mechanistic discovery to population screening), providing a practical framework for selecting context-appropriate biomarkers in radon research.

As shown in Figure 5, a decision pathway approach can steer the selection and use of radon-related biomarkers, considering the study context, analytical feasibility, and intended application. The framework connects mechanistic discovery, population monitoring, and clinical implementation by aligning biomarker categories—such as internal dose, biologically effective dose, or early biological effects—with practical factors like cost, throughput, and infrastructure. This operational model offers a structured roadmap for translating biomarker research into real-world risk assessment and public health initiatives.

Overall, cytogenetic and oxidative stress markers are readily deployable biomarkers (low-hanging fruit) because of their validation and scalability. DNA repair and epigenetic markers form a promising intermediate tier but need further standardization, whereas proteomic and multi-omics approaches are still exploratory. This tiered framework guides prioritization of biomarkers for research and policy use.

#### 3.7.6. Machine Learning for Predictive Biomarker Panels

The integration of machine learning (ML) and artificial intelligence (AI) into biomarker discovery has expanded opportunities for identifying complex, non-linear connections between molecular signatures and radon exposure outcomes. Models based on transcriptomic, proteomic, and methylation data have shown strong predictive performance in classifying exposure levels and estimating biological responses [184]. However, despite these promising findings, turning ML models into reliable, policy-relevant predictive tools requires careful methodological consideration beyond just accuracy metrics.

A major risk in high-dimensional omics and small cohort studies is overfitting, where a model learns dataset-specific noise or random fluctuations instead of true biological patterns related to exposure [185]. This issue is especially severe when the number of molecular features greatly exceeds the number of samples, a common scenario in radiation biomarker research [186]. Without thorough data partitioning methods like nested cross-validation, regularization techniques (e.g., LASSO, ridge regression), and validation using external cohorts, the predictive accuracy may be overestimated, and generalizability hindered. Recent reviews in environmental health informatics highlight that the absence of external validation continues to be a major barrier preventing the incorporation of omics-based models into epidemiological and regulatory contexts [187,188]. To develop credible and transferable models, external validation using independent populations is crucial. Ideally, models trained on one cohort (e.g., miners or residents in high-radon areas) should be tested on separate populations to assess their stability across different demographic, environmental, and lifestyle contexts [189]. This process helps identify confounders such as smoking or co-exposure to particulates that could bias the model outputs if not properly accounted for. Therefore, multi-center and cross-cohort validation are essential steps to effectively translate computational predictions into reliable risk assessments [190]. Equally important is model interpretability, a frequently overlooked aspect when applying complex ML algorithms to biological data. Transparent modeling techniques, such as feature attribution methods like SHAP (Shapley Additive Explanations) or permutation importance, allow researchers to connect predictions to specific genes, metabolites, or methylation sites [191]. This interpretability is crucial for ensuring biological plausibility and for integrating ML-based biomarkers into policy-relevant frameworks [192]. Ultimately, ML and AI models in radon biomarker research should achieve a balance between predictive accuracy, transparency, and generalizability. Forming multi-center collaborations, using standardized analytical pipelines, and openly reporting model performance metrics—including external validation and feature explainability—are essential steps toward their acceptance in public health decision-making regarding risk coefficients.

### 3.8. Synthesis and Translational Outlook

#### 3.8.1. Summary of Key Biomarker Classes and Their Utility

**Figure 6:**
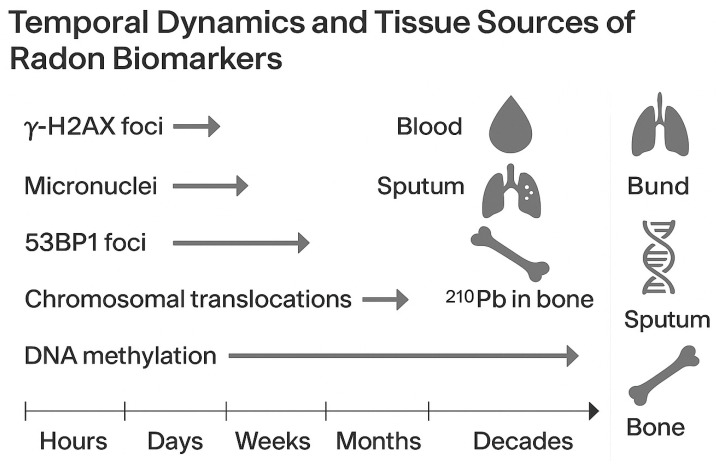
Timeline showing the persistence of key radon biomarkers, from short-term DNA damage to long-term cumulative exposure indicators across different biological tissues.

Seven decades of research have mapped a structured continuum of radon biomarkers, each representing a specific step in the pathway from exposure to disease. Internal dose biomarkers like ^210^Pb and ^210^Po in bone, teeth, and blood offer long-term records of cumulative exposure, which are essential for retrospective dose reconstruction in occupational and residential environments [193]. Biologically effective dose biomarkers, such as chromosomal aberrations, micronuclei, and γ-H2AX foci, reflect the immediate genetic damage caused by alpha-particle irradiation and are vital for biodosimetric monitoring. Additionally, early biological effect biomarkers, including gene mutations, DNA methylation changes, miRNA expression, and proteomic profiles, reveal the molecular processes that lead from DNA damage to early carcinogenic transformation. Figure 6 presents a timeline of biomarker changes, ranging from short-lived signals like γ-H2AX foci (hours to days) to mid-term indicators such as micronuclei and 53BP1 foci (weeks to months) and long-lasting signatures like chromosomal translocations or ^210^Pb accumulation (years to decades). This chronological breakdown emphasizes how transient and persistent biomarkers complement each other in monitoring both recent and past exposures. Additionally, Table 7 provides a summary of essential features, including sample sources, biological half-lives, specificity, and detection methods.

#### 3.8.2. Closing the Gaps: From Detection of Risk Reduction

Although significant progress has been made, translating biomarker discoveries into effective risk reduction strategies is still unfinished. Conventional radon risk models, mainly based on environmental and epidemiological data, do not account for individual biological differences. Using validated biomarkers allows researchers to enhance exposure–response models with greater detail, boosting their accuracy and predictive ability. For example, incorporating cytogenetic endpoints such as stable chromosomal translocations or DNA methylation signatures into dose–response models has allowed for recalibrating radon risk coefficients in both miner and residential cohorts [118,123]. Likewise, multi-omic biomarkers—which combine genetic polymorphisms (such as XRCC1 and OGG1), miRNA signatures, and inflammatory cytokines—are now being used to account for biological sensitivity, a factor not included in traditional models. These improvements can significantly decrease uncertainty in risk estimates, especially in the low-dose range (<200 Bq/m^3^) that is most relevant to the general public. Furthermore, biomarker-informed risk stratification provides tangible benefits for targeted mitigation efforts. By detecting individuals or communities showing molecular signs of ongoing DNA damage or inflammation, policymakers can focus remediation on high-risk dwellings and occupational settings. This strategy shifts biomonitoring from a passive diagnostic method to an active, proactive tool for precision prevention, where biological evidence directly guides exposure management and public health interventions.

Integrating validated biomarkers into regulatory frameworks, such as the WHO Environmental Health Criteria and the Euratom Basic Safety Standards (2013/59) [197], offers a practical route towards biologically informed radon protection. Biomarkers such as γ-H2AX and 53BP1 can quantify biologically effective dose, while DNA methylation signatures in AHRR, F2RL3, or RASSF1A provide long-term indicators of cumulative exposure. Inflammatory and oxidative stress markers, such as IL-6, TNF-α, and 8-oxo-dG, can complement physical dosimetry by revealing ongoing biological responses.

A tiered integration model could implement these endpoints within existing frameworks. Tier 1 biomarkers, such as exhaled ^210^Po and plasma 8-oxo-dG, could aid widespread community screening. Tier 2 biomarkers, like γ-H2AX and targeted methylation panels, might be useful for occupational or high-exposure groups. Tier 3 multi-omic panels could inform dose–response adjustments and epidemiological models.

Implementation would require standardized assays, international reference laboratories, and harmonized pipelines to ensure reproducibility. Integrating these tools into WHO Euratom monitoring networks would align biological evidence with exposure data, transforming radon control from a purely environmental approach to a biologically responsive risk management system.

## 4. Conclusions

The integration of validated radon biomarkers into public health initiatives represents a significant advancement in exposure prevention and the reduction of lung cancer risk. Traditional radon monitoring, which primarily measures environmental levels, should shift towards a biologically informed surveillance approach that combines environmental data with molecular biomarkers. Boimaker-guided screening can assist in identifying individuals or communities displaying early biological responses to radon exposure, such as increased γ-H2AX foci, DNA methylation of TP53 or CDKN2A, or circular miRNA changes. This enables timely interventions. In high radon areas, national authorities could adopt biomarker panels into standard radon assessment and health monitoring efforts, aligning these with frameworks such as those established by the WHO and ICRP. Using non-invasive biomarkers from blood or sputum could facilitate large-scale screening that is both feasible and cost-effective. In the future, machine learning-based multi-omic models will enable personalized risk assessments, helping to direct targeted remediation and early cancer detection. Integrating biomarker evidence into policy connects scientific research with regulation, transforming radon mitigation from population-based control to precision public health.

## Figures and Tables

**Table 1:** Comparative characteristics of internal dose and biologically effective biomarkers in radon research.

Parameter	Biomarkers of Internal Dose	Biomarkers of Biologically Effective Dose	References
Definition/Purpose	Reflects the quantity of radon progeny or their radioactive decay products retained within the body, integrating exposure over time.	Represents the biological damage caused by alpha-particle interactions, quantifying the extent of molecular and cellular injury.	[32,33]
Representative Biomarkers	^210^Pb and ^210^Po in bone, teeth, or hair; radon progeny activity in air or exhaled breath; and body burden estimates from biokinetic models.	Chromosomal aberrations (dicentrics, translocations), micronuclei in peripheral blood lymphocytes, *γ*-H2AX and 53BP1 foci (DNA double-strand breaks) 8-oxo-dG (oxidative DNA damage) Comet assay (DNA strand breaks).	[15,19,22]
Sample Type	Solid tissues (bone, teeth), soft tissues, hair, nails, exhaled air, or environmental filters.	Peripheral blood lymphocytes, sputum bronchial epithelial cells, and cultured cell lines.	[22,34]
Temporal sensitivity/Half-Life	Long-term integrators: ^210^Pb (22.3 years), ^210^Po (138 days). Reflect cumulative exposure across years or decades.	Reflect short-term or recent exposure: *γ*-H2AX foci persist 6–48 h; chromosomal translocations stable for years; oxidative lesions persist days to weeks.	[10,21]
Biological Specificity	Low to moderate; indicates exposure magnitude but not necessarily biological damage; influenced by metabolism and excretion.	High: Directly linked to cellular damage and biological effect, correlating with carcinogenic mechanisms.	[22,35]
Detection Methods	Gamma or alpha spectrometry (for ^210^Pb, ^210^Po); liquid scintillation counting, and ICP-MS; biokinetic modeling software (e.g., ICRP Legget models).	Cytogenetic assays (CBMN, FISH); fluorescence microscopy for *γ*-H2AX and 53BP1 foci, comet assay; ELISA or HPLC for 8-oxo-dG.	[19,33]
Strengths	Provides integrated, long-term exposure history; suitable for retrospective dose assessment; not influenced by short-term fluctuations.	Detects biologically meaningful effects; high sensitivity; allows dose–response modeling and mechanistic linkage to carcinogenesis.	[32,36]
Limitations	May not represent current biological effect; complex sampling (bone, teeth); influenced by diet, metabolism, and clearance.	Some markers (*γ*-H2AX, comet) are transient, have interindividual variability, and are confounded by smoking and other genotoxins.	[33,37]
Application Context	Long-term risk estimation; occupational and residential retrospective studies; model calibration.	Mechanistic and epidemiological research; validation of low-dose effects; biomonitoring of high populations.	[22,36]

**Table 2:** Comparative characteristics of key DNA-damage biomarkers under low-dose radon exposure.

Biomarker/Assay	Damage Type Captured	Temporal Behavior	Sensitivity to Dose Rate	Persistence/Repair Profile	Best Suited for	Key References
γ-H2AX/53BP1 foci	Double-strand breaks (clustered)	Peaks within hours, resolves in 1–2 days	Moderate	Transient unless repair is incomplete	Acute/short-term exposure assessment	[14,58,76,77]
Comet assay	Single/double-strand breaks, oxidative lesions	Immediate, reversible	High	Rapidly repairable	Dose–response at low/moderate exposures	[78,79,80]
Micronucleus (CBMN)	Misrepaired/unrepaired chromosomal breaks or losses	Appears after one cell cycle	Low–moderate	Stable marker of genomic instability	Chronic exposure monitoring	[52,81,82]
FISH translocation	Stable structural chromosome rearrangements	Long-term	Low	Highly persistent	Cumulative dose estimation	[83,84]

**Table 3:** Comparative overview of biomarkers and confounding factors.

Biomarker Type	Representative Assay	Primary Confounders	Effect Confounders	Key References
*γ*-H2AX, 53BP1 Foci	Immunofluorescence microscopy	Age, circadian medical imaging	High intra-individual variability; repair kinetics mask chronic exposure	[19]
Micronucleus (CBMN)	Cytokinesis-block assay	Smoking, alcohol, and nutritional status	Smoking elevated baseline frequency; antioxidants suppress formation	[107]
DNA Methylation	Infinium 450k/EPIC arrays	Age, inflammation, diet, and co-exposure	Global hypomethylation with ageing and smoking alters CpG methylation	[118]
Oxidative Stress (8-oxo-dG)	ELISA/HPLC	Tobacco, obesity, and physical activity	Elevated by smoking and metabolic stress; transient response	[114]
DNA Repair Polymorphisms	XRCC1, OGG1, XRCC3 genotyping	Ethnicity, age, radiation dose	Genetic heterogeneity modulates biomarker response	[115]
Cytokines/Proteins	Multiplex immunoassay	Infection, inflammation, and BMI	Chronic inflammation obscures exposure-related effects	[70]

**Table 4:** Comparative overview of biomarker applications in occupational vs. residential radon-exposed populations.

Exposure Context	Representative Cohorts/Study	Sample Type	Biomarker Class	Main Findings	Key References
Uranium miner (Colorado Plateau, USA)	Historical U.S Public Health Service cohort	Peripheral blood lymphocytes (PBLs)	Cytogenetic (Cas, dicentrics, rings)	Clear dose–response relationship between cumulative exposure (WLM) and chromosomal aberrations; early confirmation of radon’s genotoxicity	[26,124,140,141]
Wismut uranium miners (Germany)	Wismut cohort follow-up	Blood Serum	Stable chromosomal translocations (FISH)	Translocation frequency remained elevated decades after exposure; FISH validated as a retrospective biodosimeter	[123,142,143]
Chinese tin miners	Yunnan Tin Mining cohort	Blood lymphocytes	Micronucleus (CBMN)	Increased micronucleus frequency and DSB markers with cumulative WLM; persistent DNA repair signaling	[144,145,146,147]
Fluorspar miners (Newfoundland, Canada)	Newfoundland Fluorspar cohort	Blood and sputum	CAs; oxidative stress (8-oxo-dG)	Long-term persistence of stable translocations 20+ years post exposure; used for retrospective dose assessment	[148,149,150]
Residential Exposure (Iowa Radon Study, USA)	Iowa Lung Cancer Study	Blood lymphocytes	Micronucleus (CBMN); comet assay	Elevated DNA strand breaks and micronuclei at ≥150 Bq/m^3^; correlation with long-term indoor radon	[52,58,62,151]
European pooled residential studies	13 European case–control studies	Blood, sputum	Cytogenetic and epigenetic markers	Linear increase in lung cancer risk with indoor radon; confirmed by epigenetic alterations (global DNA hypomethylation)	[99,118,152,153]
Swedish residential radon cohort	Swedish Lung Cancer Study	Blood DNA	DNA methylation (CpG-specific)	Promoter hypomethylation of tumor suppressor genes (e.g., RASSF1A, CDKN2A) associated with higher indoor radon	[1,3,154]
Central European population (multi-country)	Prospective biomarker cohort	Serum, plasma	Cytokines, miRNAs, proteomics	Elevated IL-6, TNF-∝ and miR-21 in high-radon areas (<100 Bq/m^3^; early inflammation and oxidative response signatures	[155,156,157]
Radon + Smoking Interaction (mixed populations)	Pooled miner and residential datasets	Lung tumor tissue	TP53, KRAS mutation profiling; miRNAs	TP53 G → A transitions overlap in radon and smoke-induced cancers; supports the multiplicative model	[158,159,160]

**Table 5:** Practical and translational feasibility of major biomarker classes.

Biomarker Class	Measurement Platform	Infrastructure Needs	Throughput	Relative Cost	Deployment Feasibility	References
Cytokines/Autoantibodies	ELISA/Multiplex bead array	Bench-top immunoassay reader	High	Low	Suitable for point of care or regional labs	[179,180]
DNA Damage (γ-H2AX, Micronucleus, FISH)	Immunofluorescence/Flow cytometry	Basic cytogenetic or imaging lab	Moderate	Moderate	Centralized biomonitoring facilities	[179,181,182]
Epigenetic Markers (DNA methylation, miRNA)	qPCR/Targeted NGS	Sequencing core lab	Moderate	Moderate–high	Central research or diagnostic labs	[179,182]
Proteomics/Metabolomics	LC-MS/MS	High-end mass spectrometry	Low	High	Specialized research centers only	[179,181]
Multi-omics integration	LC-MS/MS + NGS + Bioinformatics pipeline	Large-scale multi-omic infrastructure	Low	Very High	Limited to research consortia or specialized centers	[179,181,183]

**Table 6:** Decision-oriented synthesis of major biomarker classes in radiation research.

Biomarker Class	Biological Relevance	Technical Feasibility	Translational Readiness	Current Status
Cytogenetic markers (micronuclei, chromosomal aberrations)	High	High	High	Well-suited for occupational and population monitoring
DNA damage response (γ-H2AX, 53BP1 foci)	High	Moderate	Moderate	Mechanistically specific; requires standardization and automation
Oxidative Stress (8-oxo-dG)	Moderate	High	High	Easily measurable; promising for biomonitoring, though not radon-specific
Epigenetic Markers (DNA methylation, mRNA)	High	Moderate	Moderate	The emerging class requires longitudinal validation and population data
Proteomic/metabolomic markers	Moderate	Low	Low	Biologically informative but currently limited by variability and cost
Multi-omics panels	Very high	Low	Exploratory	Valuable for discovery but not yet scalable for field deployment

**Table 7:** Summary of Major Radon Biomarker Classes: Temporal Persistence, Sample Sources.

Biomarker Class	Representative biomarkers	Biological Half-Life Persistence	Primary Sample Source	Detection/ Quantification Methods	Biological Relevance and Translational Utility	References
Internal Dose Biomarkers	^210^Pb, ^210^Po accumulation; radon progeny in bone, teeth, and blood	Long-term (years–decades)	Bone, teeth, blood	Gamma spectrometry, alpha spectrometry, ICP-MS	Reflect cumulative radon deposition and inhalation dose; ideal for retrospective exposure reconstruction	[194,195,196]
Cytogenetic Biomarkers (Biologically Effective Dose)	Dicentrics, rings, translocation (FISH); micronuclei (CBMN)	Weeks to years (depending on stability)	Peripheral blood lymphocytes (PBLs)	FISH, CBMN assay, automated metaphase scoring	Quantify alpha-particle-induced DNA misrepair and chromosomal instability, useful for dose estimation and biological monitoring	[70]
fDNA Damage and Repair Biomarkers	*γ*-H2AX foci, 53bP1, comet assay parameters	Hours–days (transient)	PBLs, bronchial epithelial cells	Immunofluorescence microscopy, flow cytometry, and comet electrophoresis	Indicate recent double-strand break induction and repair kinetics; sensitive to acute or ongoing exposure	[70]
Oxidative Stress Biomarkers	8-oxo-dG, lipid peroxidation products (MDA), and antioxidant enzyme levels	Days–weeks	Blood, urine, sputum	HPLC, LC-MS/MS, ELISA	Reflect indirect DNA and lipid damage from *α*-induced reactive oxygen species (ROS); bridge radiation and inflammatory pathways	[70]
Epigenetic Biomarkers	Global and CpG-specific DNA methylation (e.g., TP53, RASSF1A); histone acetylation/methylation marks	Weeks–months	Whole blood, sputum, lung tissue	Bisulfite sequencing, methylation arrays, ChIP-seq	Capture early molecular imprint of chronic exposure; potential early detection tools in non-invasive samples	[70]
miRNA Biomarkers	Circulating miR-21, miR-33a, miR-155	Weeks–months	Serum, plasma, sputum	qRT-PCR, RNA-Seq	Regulates post-transcriptional stress response; non-invasive and stable; candidates for early disease prediction	[70]
Genetic Expression Transcriptomic Biomarkers	GADD45A, NFE2L2, TP53, HMOX1 activation	Hours–days (dynamic)	Blood, bronchial epithelial cells	RNA-Seq, qPCR, microarrays	Identify activation of DNA repair, oxidative stress, and inflammation pathways; mechanistic insight into radon-induced carcinogenesis	[70]
Proteomic biomarkers	Cyrokines (IL-6, TYNF-*α*, IL-8) growth factors; autoantibodies to TAAs	Weeks–months	Serum, plasma	ELISA, LC-MS/MS antibody arrays	Reflect systematic inflammatory and immune response to exposure; useful for screening or follow-up in epidemiology studies	[70]
Multi-Omic/Integrated Panels	Combined methylation + miRNA + protein signatures	Variable (multi-scale)	Blood, plasma, saliva	Multi-omic integration (ML-based)	Offer personalized exposure and risk prediction, and bridge internal dose and disease outcomes in precision public health frameworks	[70]

## Data Availability

No new data were created or analyzed in this study. Data sharing is not applicable to this article.

## Supplementary Materials

The following supporting information can be downloaded at https://www.mdpi.com/article/10.3390/ijms27104391/s1.
